# pH-tuned reversible self-assembly of Janus particles for enhanced Raman imaging and sensing

**DOI:** 10.1007/s00216-025-05887-z

**Published:** 2025-05-03

**Authors:** Maria Iftesum, Mohan Kumar Dey, Alisha Prasad, Jin Gyun Lee, Ram Devireddy, Bhuvnesh Bharti, Manas Ranjan Gartia

**Affiliations:** 1 Department of Mechanical and Industrial Engineering, Louisiana State University, Baton Rouge, LA 70803 USA; 2 Catalent Pharma, St. Petersburg, FL 33716 USA; 3 Cain Department of Chemical Engineering, Louisiana State University, Baton Rouge, LA 70803 USA

**Keywords:** Janus particles, Raman spectroscopy, SERS, PH-tuning, Self-assembly, FDTD, Biosensors

## Abstract

The controlled self-assembly of Janus particles via pH modulation offers an effective strategy for optimizing surface-enhanced Raman scattering (SERS) and Raman signal amplification. These asymmetric microparticles enable precise spatial organization, influencing plasmonic coupling and electromagnetic field distribution critical for SERS enhancement. Here, we investigate the pH-mediated self-assembly of Janus particles and its impact on their optical sensing performance. By adjusting solution pH, we modulate electrostatic interactions that govern particle aggregation and structural formation, leading to tunable hotspots for Raman signal enhancement. Scanning electron microscopy (SEM) characterizes the resulting nanostructures, while Raman imaging and SERS measurements assess the enhancement capabilities across different pH conditions. Our findings demonstrate that pH-mediated self-assembly plays a pivotal role in optimizing interparticle spacing and plasmonic interactions, yielding significantly amplified Raman signals. This approach provides a versatile and reproducible method for engineering SERS-active substrates, advancing their application in bioanalytical sensing, molecular diagnostics, and environmental monitoring.

**Graphical abstract:**

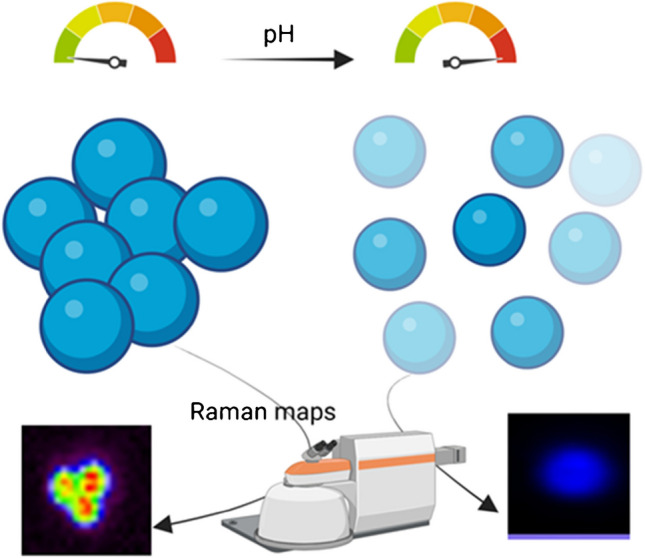

**Supplementary Information:**

The online version contains supplementary material available at 10.1007/s00216-025-05887-z.

## Introduction

Surface-enhanced Raman spectroscopy (SERS) has emerged as a powerful analytical technique for detecting and characterizing molecules at extremely low concentrations, owing to its ability to amplify weak Raman signals through plasmonic enhancement mechanisms [1–3]. The development of advanced nanostructures has played a pivotal role in optimizing SERS performance [4–8], with particular emphasis on improving sensitivity, reproducibility, and molecular specificity. Among these, Janus particles [9–13]—bifunctional or asymmetrically structured nanoparticles—represent a promising frontier in SERS innovation [12, 14–20].

Janus particles are uniquely engineered to exhibit distinct physical or chemical properties on each of their hemispheres, enabling multifunctionality and tunability in their interaction with analytes and electromagnetic fields [16, 17, 20]. This asymmetric design provides several advantages over traditional SERS substrates, including the ability to modulate local surface plasmon resonances [13], enhance molecule-particle interactions [12, 21], and enable directional binding or orientation of target molecules [22, 23]. Moreover, their versatile surface chemistries and potential for self-assembly facilitate the design of tailored platforms for diverse applications, ranging from biomedical diagnostics to environmental sensing [13, 24–26]. Despite their potential, the application of Janus particles in SERS remains an area of active exploration. This study aims to investigate the synthesis, characterization, and performance of Janus particles as SERS substrates, focusing on their ability to achieve signal modulation under different pH conditions, and understanding the mechanism using theoretical modeling. The ability to control particle aggregation is fundamental to numerous applications in materials science, chemistry, and biology, including drug delivery, colloidal stabilization, and the design of functional nanostructures [27–30]. Among the various strategies to modulate particle behavior, pH-responsive systems offer a dynamic and versatile approach [31–33], particularly for particles that exhibit chemical or structural asymmetry, such as Janus particles [34, 35]. Janus particles, characterized by their distinct surface chemistries or physical properties on different regions, have garnered significant interest for their ability to self-assemble into hierarchical structures with tunable properties [20, 36].

However, several challenges hinder the widespread adoption of Janus particle–based SERS. Reproducibility and quantification remain critical concerns due to inherent variations in hotspot formation and fluctuating signal intensity across different batches and experimental conditions, which can undermine quantitative analyses; recent studies have addressed these issues by introducing internal standards and robust calibration methods to enhance measurement reliability and reproducibility [16]. Additionally, the scalability of fabrication remains a major challenge, particularly in producing uniform sub- 500 nm Janus particles with consistent properties. Although advances in microfluidic synthesis and template-assisted methods show promise, further refinement is necessary to achieve high-throughput production without compromising quality [37]. Another key limitation is signal consistency, as the formation of plasmonic hotspots critically depends on controlled aggregation and precise interparticle spacing, leading researchers to explore integrated hotspot platforms and advanced assembly techniques that promote uniform hotspot distribution for enhanced sensitivity and stability [16, 38]. Furthermore, the biocompatibility and environmental impact of metallic Janus particles pose serious concerns, especially for in vivo applications and sustainable technologies; recent investigations have focused on developing biodegradable and non-toxic alternatives through the incorporation of biocompatible materials or surface modifications designed to minimize environmental risks [39, 40]. Addressing these challenges is crucial for advancing Janus particle–based SERS in biosensing, diagnostics, and environmental applications, and will require a multidisciplinary approach that integrates innovations in materials science, chemistry, and engineering.

pH tuning provides a unique mechanism for directing the aggregation of Janus particles by influencing interparticle forces such as electrostatic, van der Waals, and hydrogen-bonding interactions [11, 41]. Through strategic design, the surface properties of Janus particles can be engineered to exhibit pH-sensitive functionalities, such as protonation or deprotonation of specific groups, thereby enabling reversible aggregation and disassembly [35, 42]. This pH-dependent behavior is particularly advantageous for applications requiring precise control over particle aggregation states, such as stimuli-responsive materials, sensing, and drug delivery systems [43].

In this work, we introduce a practical and tunable approach to modulate SERS signals using Janus particles that self-assemble in response to pH changes. While most earlier studies have focused on static or pre-assembled SERS substrates [16], our method enables dynamic control over particle aggregation and plasmonic hotspot formation by simply adjusting the solution pH. This makes the system not only user-friendly but also highly adaptable to diverse biological or environmental settings. At the same time, there is still a limited understanding of how pH affects the aggregation of Janus particles, particularly given their asymmetric surface chemistry. To gain a deeper understanding, we employed Raman spectroscopy to monitor changes in the signal from protein-functionalized Janus particles, in conjunction with electromagnetic simulations. This approach enabled us to understand how surface charge heterogeneity and chemical groups affect aggregation behavior, interparticle spacing, and field enhancement. These findings highlight the potential of Janus particles as responsive and flexible SERS platforms for biosensing, environmental monitoring, and other real-world analytical applications [20, 44].

Despite its potential, the fundamental understanding of pH-driven aggregation mechanisms in Janus particles remains limited, particularly with respect to how asymmetry in surface properties influences aggregation dynamics and final assembly structures. We investigate how pH influences the aggregation behavior of Janus particles by using Raman spectroscopy to measure the signal from the protein attached to these particles. This study aims to uncover the fundamental role of surface charge heterogeneity and chemical functionality in governing aggregation thresholds and morphologies. By elucidating how pH modulates Janus particle aggregation, these findings could pave the way for designing advanced materials with tailored aggregation behaviors, offering new opportunities in environmental remediation, biomedicine, and nanotechnology.

## Materials and methods

### Fabrication of Janus particles

Polystyrene (PS) microspheres (2.0 μm diameter, Magsphere Inc.) were functionalized with carboxylate groups, imparting a negative surface charge (zeta potential at pH 7 was − 47 mV). Zeta potential measurements were performed using a Univette cuvette (Anton Paar), with electrophoretic mobility determined in water (0.01 wt%) by applying a 10-V direct current across the dispersion. Sub-monolayers were prepared using a convective assembly method [45, 46]. To remove surfactants, the particles were washed three times with water and concentrated to 10 wt%. After sonication, 10 μL of the particle suspension was pipetted onto the substrate slide at the contact point with the deposition slide, forming a thin, uniform line along the contact edge. The particles were coated with 10 nm of chromium (deposition rate: 0.5 nm/s), followed by 20 nm of gold (deposition rate: 0.1 nm/s) under vacuum (1 × 10^−6^ Torr) using a thermal evaporator (Thermionics Laboratory VE- 90). Coated particles were removed from the slides by gentle scraping with a spatula, resuspended in water, and transferred to a microcentrifuge tube for storage. To disperse aggregates, the particles were sonicated for ~ 30 s prior to experiments. All experiments were conducted using ultrapure water [20] (resistivity: 18.2 MΩ∙cm).

### PEG functionalization and self-assembly of Janus particles

To examine the influence of polyethylene glycol (PEG) functionalization on self-assembly, a comparative study was conducted using Janus particles with and without PEG functionalization in the presence of a lysozyme protein solution. Two distinct procedures were employed to fabricate PEG-functionalized Janus particles. In the first method, the PS particles (concentration = 0.01 wt%) were dispersed in a PEG aqueous solution (Alfa Aesar, molecular weight = 20,000 Da, concentration = 0.1 mg/mL) and equilibrated overnight at 25 °C. The particles were then thoroughly rinsed with water to remove any unbound components. The resulting Janus particles (concentration = 0.01 wt%) were re-suspended in a 0.1 mg/mL lysozyme aqueous solution and equilibrated at 25 °C for 24 h for further analysis. The second method involved direct metal deposition onto PS particles before PEG functionalization. The metal-coated Janus particles were then dispersed in a PEG solution and left to equilibrate overnight. After extensive washing to remove excess PEG, the particles were re-suspended in a 0.1 mg/mL lysozyme solution, following the same protocol as in the first method.

### Electron and optical microscopy imaging

Scanning electron microscopy (SEM) (Quanta 3D DualBeam FEG FIB-SEM) was employed to examine the geometry and structural organization of Janus particles under an operating voltage of 1 kV. For SEM imaging, a glass cutter sectioned the substrate containing assembled gold-coated patchy spherical particles into smaller pieces. Brightfield microscopy was utilized to observe the surface assembly, patch morphology, and active motion of patchy spheroids using a Leica DM6 microscope. Dynamic motion of the particles was recorded at 50 frames per second using a Leica DFC9000 GTC digital camera.

### Raman spectroscopy and imaging

The Raman spectra were acquired using a Renishaw inVia Reflex system with a 50× air objective. The excitation wavelength (λ_ex_) was 532 nm, with the laser power set to 1% (0.3 mW) and an exposure time of 60 s per spectrum. Spectra were collected over a wavenumber range of 100–3200 cm^−1^. Preprocessing involved baseline correction and vector normalization. Raman imaging was conducted at a spatial resolution of 0.3 μm × 0.3 μm using the StreamHR mode with a 532 nm laser, 50× air objective, and a 1800 grating. Imaging was performed at 1% laser power (0.3 mW), with a 1-s exposure time, centered at 1000 cm^−1^.

### Finite difference and time domain (FDTD) electromagnetic simulations

The electromagnetic field of the Janus particle was simulated using Ansys Lumerical FDTD with a wavelength range of 300 to 7000 nm. A total-field scattered-field (TFSF) source was used to analyze the scattering characteristics of the microparticle, and perfectly matched layer (PML) boundaries were applied to accurately compute the absorption cross-section (also see Electronic Supplementary Material Fig. S1). The simulation grid size was set to 5 µm × 5 µm. The refractive index of gold (Au) was defined using data from Babar and Weaver, 2015 from the database [47], while polystyrene (PS) was set at 1.5698 [47]. To model the gold-coated polystyrene Janus particle, the mesh order was defined to determine material priority in overlapping regions: air (domain 1), gold (domain 2, d = 2.04 µm), and polystyrene (domain 3, d = 2.0 µm). Simulations were performed for single, two, and three Janus particle configurations, with a 30-nm gap between adjacent particles in multi-particle setups.

## Results and discussions

**Fig. 1:**
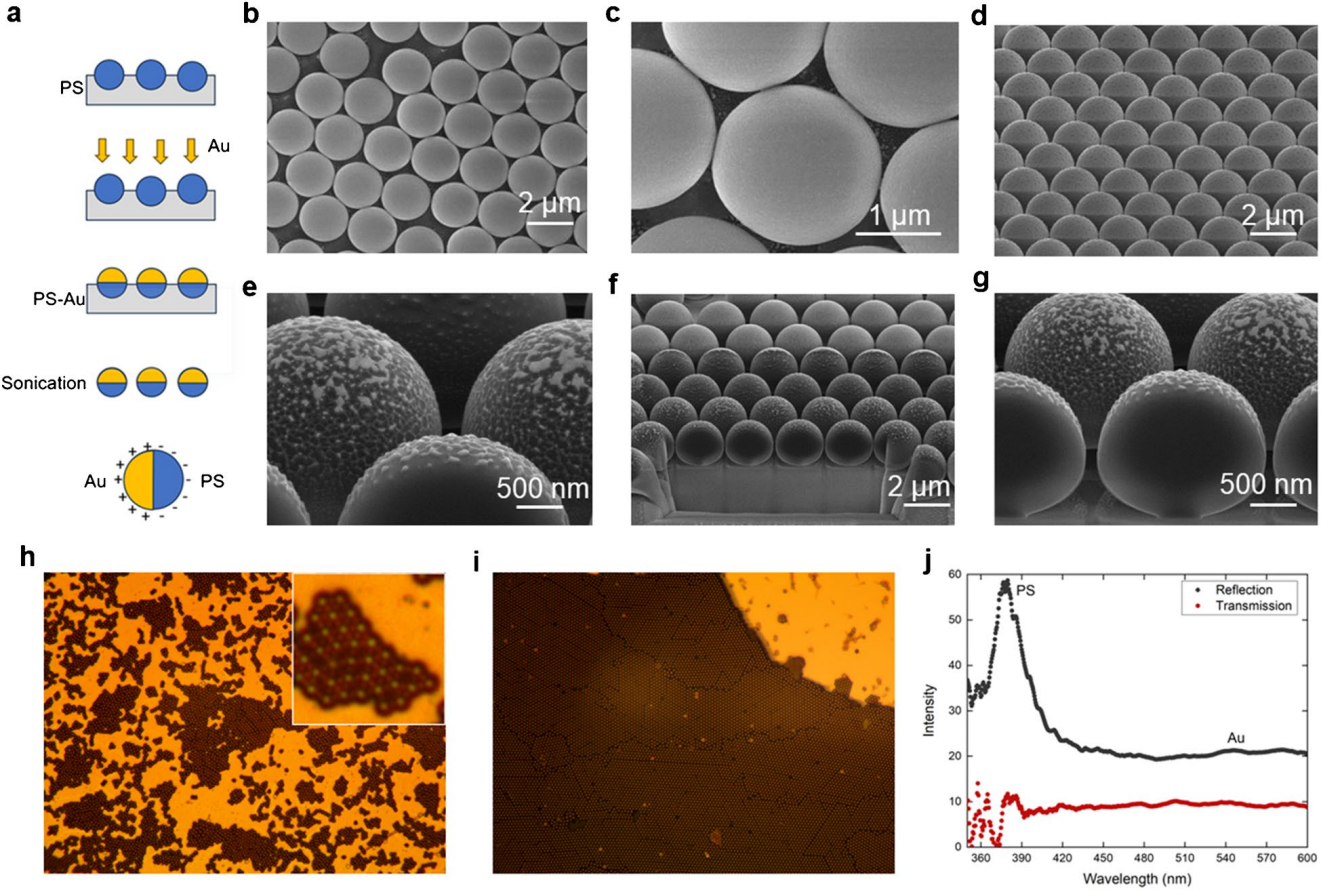
**a** Schematic illustration of the Janus particle (JP) synthesis process. **b** Low-magnification and **c** high-magnification scanning electron microscopy (SEM) images of the self-assembled polystyrene (PS) beads. **d** Top-view SEM image of JPs after metal (Au) deposition. **e** Tilted (52°) SEM view of the JPs. **f**, **g** Cross-sectional SEM images of the JPs. **h**, **i** Optical images showing large-area self-assembled PS bead arrays. **j** Reflection and transmission spectra of the JPs on a glass slide

Figure 1a schematically shows the synthesis of Janus particle. Two-micrometer polystyrene beads were self-assembled on a glass slide. Then a thin layer of gold (30 nm) was deposited to create the Janus particle. Figure 1b and c shows the top view, and Fig. 1d–g shows the cross-sectional view of the Janus particles using scanning electron microscopy (SEM). The asymmetric material arrangement (top gold layer and bottom PS layer) could be clearly seen in Fig. 1d.

**Fig. 2:**
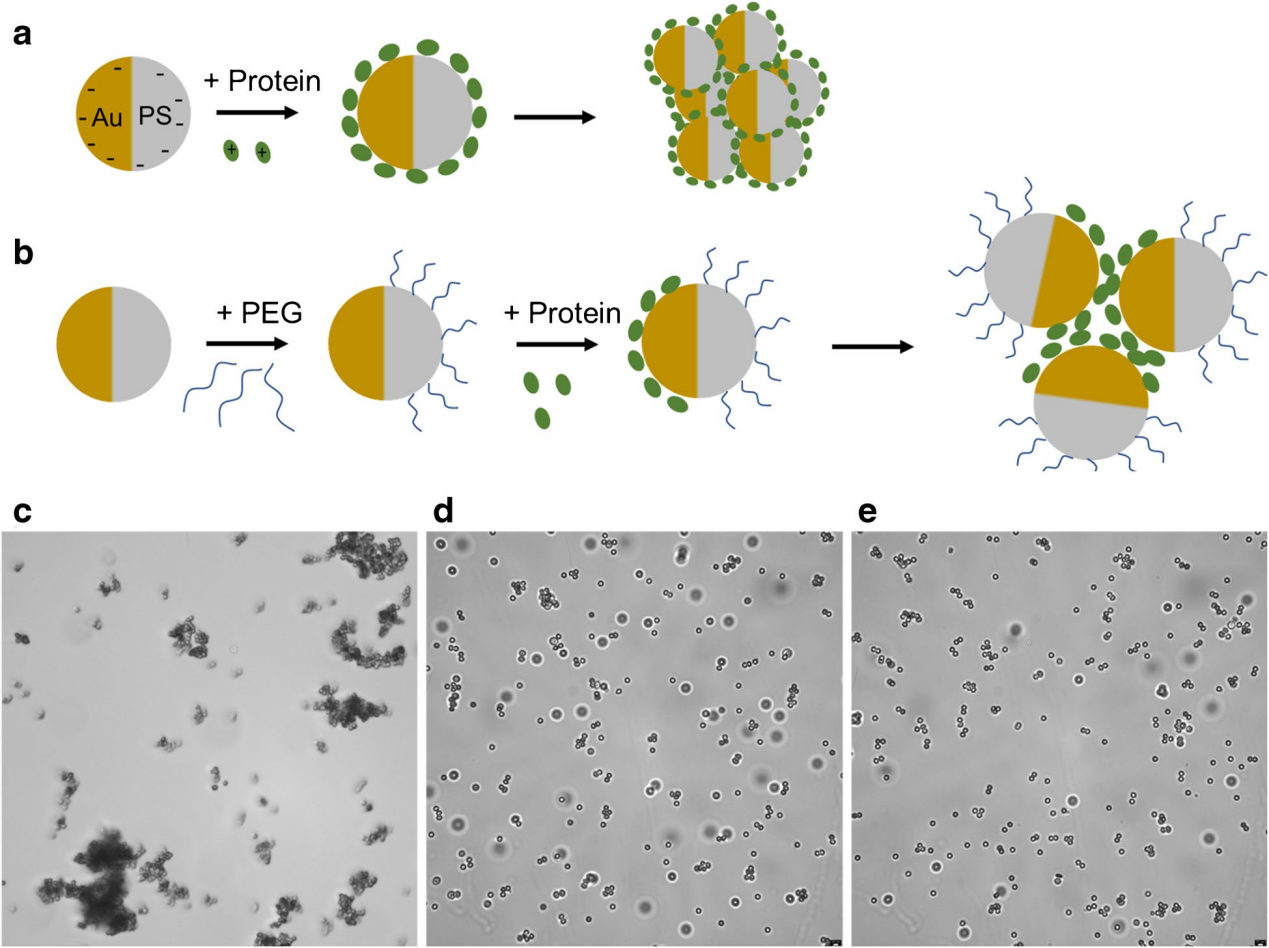
Non-PEGylated and PEGylated Janus particles. **a** Schematic representation of particle aggregation after protein incubation without PEG functionalization and **b** with PEG functionalization. Bright-field images of **c** non-PEGylated Janus particles incubated with lysozyme, **d** PEG-functionalized particles coated with 30 nm gold followed by lysozyme incubation, and Janus particles coated with metal, subsequently functionalized with PEG, and then incubated in a lysozyme protein solution

To optimize Raman signal modulation, molecules ideally need to be positioned between two metal surfaces. To achieve this configuration while preventing direct contact, polyethylene glycol (PEG) functionalization was used. A comparative study was conducted to assess the impact of PEG functionalization on self-assembly, using lysozyme as a model protein (schematically shown in Fig. 2b). As expected, Janus particle aggregation was observed in the absence of PEG functionalization (Fig. 2a and c).

Two synthesis approaches were employed for PEG-functionalized Janus particles. In the first method, 2.0 μm carboxylate polystyrene (PS) particles were dispersed in a PEG solution and allowed to settle overnight before being rinsed to remove unbound components. In this study, we used PEG with hydroxyl (-OH) terminal groups. The pKa of the -OH groups in the PEG molecule is ~ 15. Hence, these groups would not deprotonate under normal pH conditions, ranging from pH 2 to 10. Thus, the PS side of the Janus particle would remain invariant. In contrast, the gold-coated side of the Janus particles would alter with the pH, leading to the observed protein-mediated assembly and disassembly of the particle clusters.

A 10-nm Cr adhesion layer followed by a 30-nm Au layer was then deposited. The resulting Janus particles were re-suspended in a 0.1 mg/mL lysozyme solution (Fig. 2d). The second method involved direct metal deposition onto carboxylate PS particles, followed by PEG treatment and overnight stabilization. After rinsing, these particles were also re-dispersed in a 0.1 mg/mL lysozyme solution (Fig. 2e). Both approaches enabled effective PEG functionalization, enhancing interactions with lysozyme (Fig. 2d and e). This study demonstrated that the absence of PEG functionalization resulted in random aggregation of Janus particles (Fig. 2c), whereas PEG functionalization enabled controlled aggregation (Fig. 2d and e; also see Electronic Supplementary Material video).

**Fig. 3:**
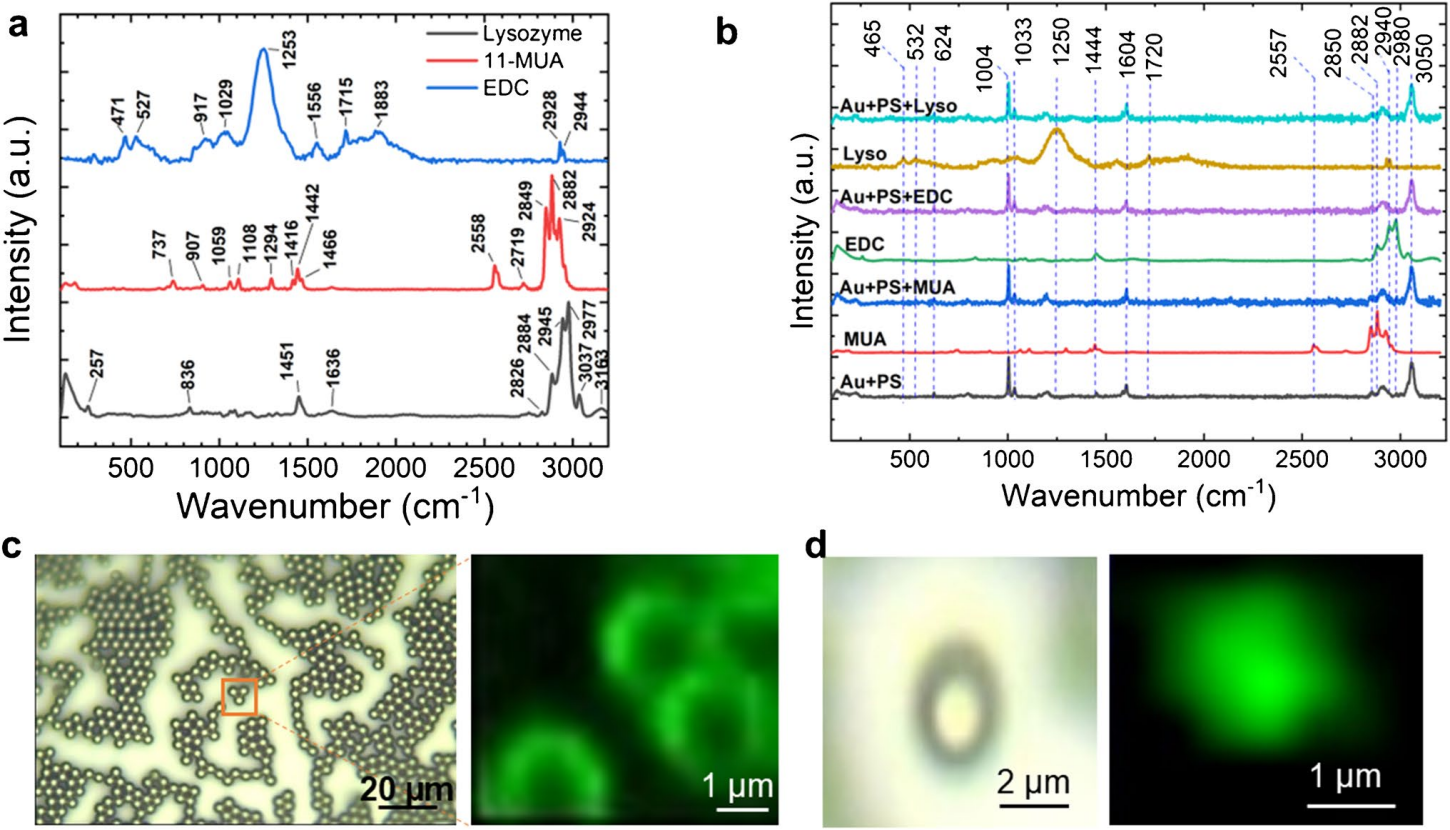
**a** Raman spectra of the molecules used in protein conjugation to JPs, showing characteristic peaks for Lysozyme, 11-MUA, and EDC. **b** Raman spectra illustrating each step of the conjugation process: JP alone (black), JP with the 11-MUA linker (blue), JP after EDC activation (purple), and JP after protein conjugation (magenta). Bright-field and corresponding Raman images at the 1005 cm⁻^1^ peak for **c** multiple JPs and **d** a single JP are shown

To further control aggregation, we functionalized the Janus particles with MUA linker molecules. MUA contains an S-H group, which exhibits a Raman vibration peak at 2549 cm⁻^1^ (Fig. 3a, red curve). After conjugation via the S-Au bond, the S-H peak disappears, confirming successful attachment (Fig. 3b, blue curve). Additionally, the presence of a peak at 1583 cm⁻^1^ (Amide II) verifies the successful conjugation of lysozyme.

Raman spectra of JP are shown in Fig. 3a, b that have undergone various functionalization and immobilization processes. The Janus nanoparticles were functionalized with 11-MUA (11-mercaptoundecanoic acid) [48], crosslinked using EDC (1-ethyl- 3-(3-dimethylaminopropyl) carbodiimide) [49], and subsequently immobilized with lysozyme [15]. The Raman spectra confirmed the characteristic molecular vibrations of each component (see Table 1 and Electronic Supplementary Material Table S1). Lysozyme exhibited distinct peaks, with one at 622 cm⁻^1^ corresponding to ring deformation, while peaks at 648 and 755 cm⁻^1^ were associated with tyrosine and tryptophan residues. Additional peaks at 1122 and 1255 cm⁻^1^ indicated C-N and C-C stretching, and a peak at 1551 cm⁻^1^ corresponded to peptide bonds (Amides III and II). For MUA, the spectrum revealed a peak at 738 cm⁻^1^ linked to anti C-C stretching, and another at 909 cm⁻^1^ associated with C-COOH stretching. Peaks at 1060 and 1290 cm⁻^1^ indicated anti C-C and anti C-O stretching with OH bending, respectively. Further peaks at 1436 and 1457 cm⁻^1^ corresponded to CH₂ bending and COO⁻ groups, while peaks at 1631 and 2549 cm⁻^1^ were attributed to C=O stretching and Amide I structures. The EDC spectrum displayed peaks at 1149 cm⁻^1^ (NH_3_⁺), 1457 cm⁻^1^ (COO⁻), and 1631 cm⁻^1^ (C=O stretching). Meanwhile, the Janus particles showed a dominant peak at 1005 cm⁻^1^, indicating C-C stretching in polystyrene. Bare PS particles exhibited peaks at 622 cm⁻^1^ (ring deformation), 1030 cm⁻^1^ (aromatic C-C stretching), 1157 cm⁻^1^ (NH₅⁺), and 1452 cm⁻^1^ (COO⁻). These spectral features confirm the successful functionalization of the particles and the presence of their respective molecular components. Figure 3c and d shows the Raman mapping images of Janus particles at a spatial pixel resolution of 300 nm × 300 nm. The Raman images were constructed at 1005 cm^−1^.

**Table 1:** Raman characteristic peaks and their assignment for the JP

Contributing group	Frequency (cm^−1^)	Assignment
Polystyrene beads [50]	622	Ring deformation
	1005	Phenylalanine
	1029	C–C stretching of aromatic ring
	1603	C = C aromatic ring stretching
	1452	C–CH stretching of aromatic ring
11-MUA [51, 52]	738	Anti C-S stretching
	909	C-COOH stretching
	1060	Anti C–C stretching
	1107	Anti C–C stretching
	1290	Anti C-O stretch and OH bending
	1436	CH_2_ bending
	1458	CH_2_ bending
	1631	Anti C = O stretch
	2549	Anti S–H stretch
	2575	Anti S–H stretch
EDC [53, 54]	827	C–C stretch
	1149	NH_3_^+^
	1454	COO − stretch
	1642	C = O stretch, α-helices, Amide I
Lysozyme [55, 56]	648	Tyrosine
	755	Tryptophan
	1122	C-N, C–C stretching
	1255	α-helices, C-N peptide bond (Amide III)
	1444	CH deformational vibrations
	1551	Amide II

By systematically analyzing the appearance and disappearance of Raman peaks, we can elucidate the conjugation of specific molecules to Janus particles. 11-MUA molecules were attached to the Janus particle surfaces via thiol (-SH) groups, which exhibit a characteristic Raman vibrational peak at 2549 cm⁻^1^. This peak is observed in neat 11-MUA solutions. Successful conjugation of 11-MUA via Au-S bonding is confirmed by the disappearance of the 2549 cm⁻^1^ peak. Subsequently, EDC crosslinking was performed by introducing a solution containing EDC and N-hydroxysuccinimide (NHS) to the 11-MUA-functionalized Janus particles. EDC activates the carboxyl (-COOH) groups on 11-MUA, while NHS facilitates the formation of reactive ester intermediates. These intermediates enable covalent bonding between the carboxyl groups of 11-MUA and the amine (+NH₂) groups of proteins. Finally, lysozyme, the protein of interest, was prepared in an appropriate buffer or solvent and added to the EDC-crosslinked Janus particles. The amine groups in lysozyme react with the activated ester intermediates on the Janus particle surfaces, forming covalent bonds and immobilizing lysozyme onto the Janus particles. The successful conjugation of lysozyme is indicated by the presence of a Raman peak at approximately 1191 cm⁻^1^, which corresponds to a characteristic peak in the raw lysozyme spectrum.

**Fig. 4:**
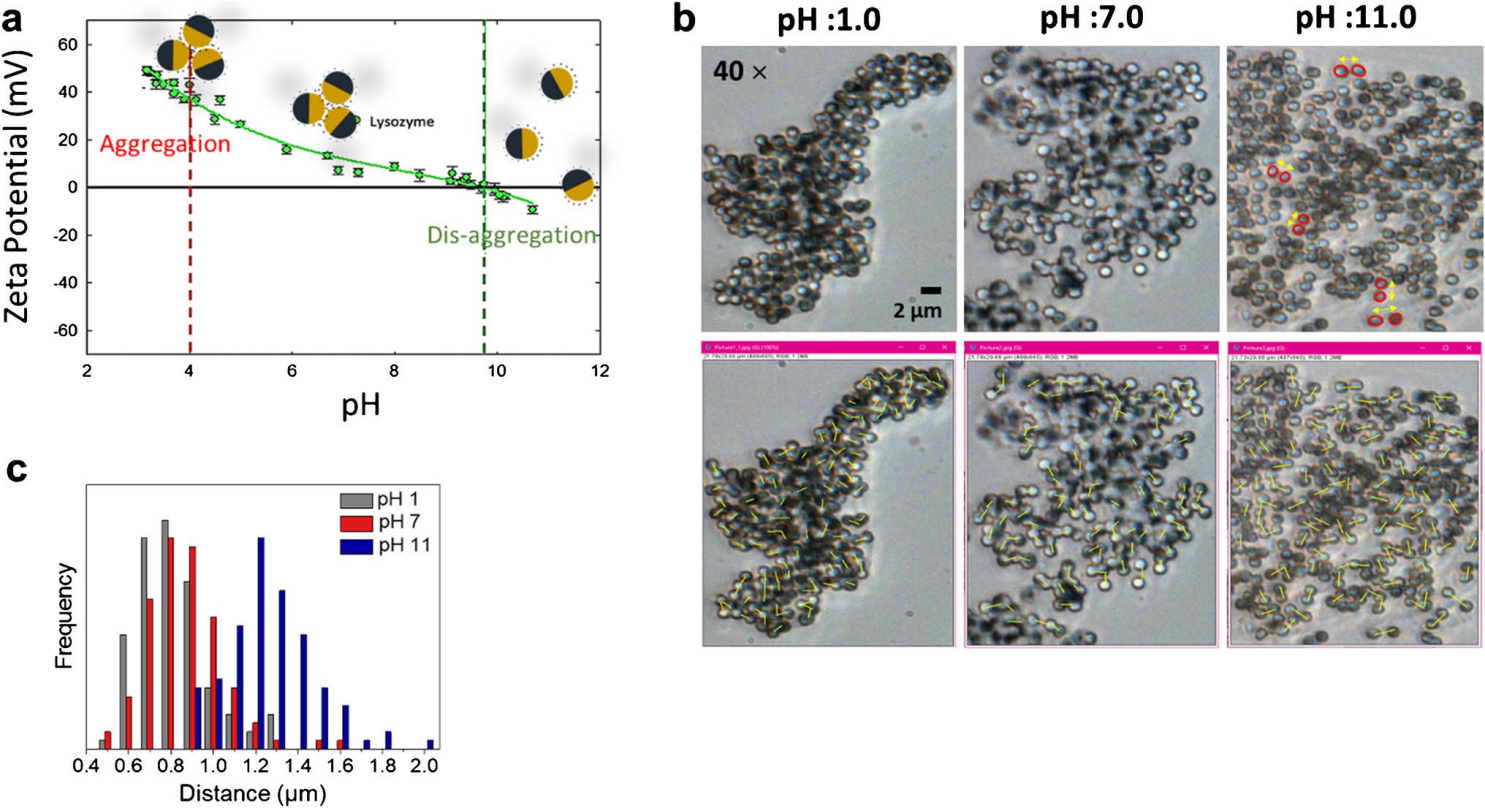
pH-Dependent Assembly of Janus Particles. **a** Zeta potential measurements of the JPs as a function of pH, illustrating changes in surface charge with varying pH levels. **b** Image-based analysis used to measure the distance between neighboring JPs. **c** Variation in the inter-particle distance across different pH conditions, highlighting the influence of pH on JP assembly

Following the successful conjugation of lysozyme to the Janus particles (JPs), we explored whether their Raman signal could be modulated by altering their aggregation behavior. The JPs demonstrated reversible aggregation and disaggregation in response to pH changes [6]. Figure 4 illustrates this phenomenon, showing that increasing the pH leads to an increase in the inter-particle distance. Specifically, at pH 1.0, the particles are closely packed, whereas at pH 11.0, the distance between neighboring particles increases significantly, as seen in Fig. 4b. This behavior is also evident in the particle distribution frequency, where a higher frequency (greater number of particles at shorter distances) is observed at low pH. In contrast, as the pH increases, the inter-particle distance increases, resulting in a lower frequency of closely spaced particles (Fig. 4c). The underlying mechanism governing this pH-dependent aggregation is closely related to the zeta potential of the particles, as shown in Fig. 4a. Zeta potential represents the electrostatic potential at the interface between the particle surface and the surrounding solution, providing insights into surface charge characteristics. The ionization state of surface functional groups, such as carboxyl or amino groups, is influenced by the pH of the solution, thereby modulating the zeta potential. At pH 4, the particles exhibit aggregation, corresponding to a zeta potential of approximately 40 mV. In this state, the attractive forces between particles dominate, leading to particle clustering. However, as the pH increases, the zeta potential approaches neutrality, reducing the electrostatic attraction and increasing repulsive interactions between similarly charged particles. Consequently, lower pH levels promote particle aggregation, while higher pH levels facilitate their disaggregation through enhanced electrostatic repulsion.

**Fig. 5:**
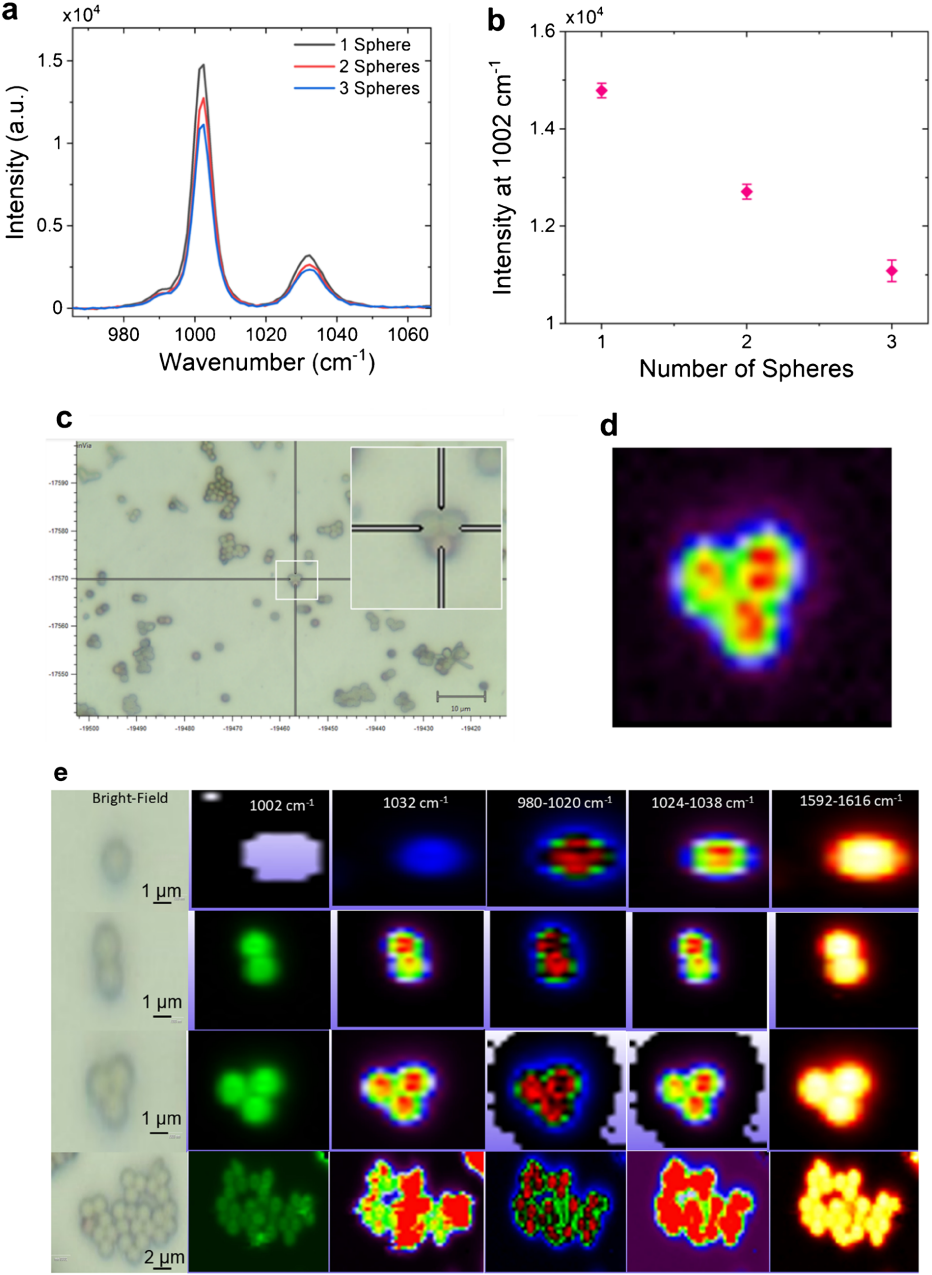
Raman spectra and Raman mapping of assembled Janus particles (JPs). **a** Comparison of Raman intensity at 1002 cm⁻^1^ and 1035 cm⁻^1^ for assemblies of a single, two, and three JPs. **b** Raman intensity at 1002 cm⁻^1^ as a function of the number of JPs. **c** Bright-field image and **d** corresponding Raman mapping of a three-JP assembly at 1002 cm⁻^1^. **e** Bright-field images and respective Raman mappings for assemblies of one, two, three, and multiple JPs, recorded at 1002, 1032, 980, 1024, and 1592 cm⁻^1^

To investigate the effect of aggregation on the Raman signal, we performed Raman spectroscopy and the corresponding Raman mapping on a single, two, three, and multiple JPs. Figure 5a shows the Raman spectrum obtained from one (black), two (red), and three (blue) JPs. We observed a decreasing trend in Raman intensity (at 1002 cm^−1^) with increase in the number of spheres (Fig. 5b). Figure 5c shows the bright-field and Fig. 5d shows the corresponding Raman mapping image of three JPs. The bright-field and corresponding Raman mapping for one, two, three, and multiple JPs taken at 1002, 1032, 980, 1024, and 1592 cm^−1^ is shown in Fig. 5e.

The decrease in Raman intensity with an increasing number of spheres can be attributed to two main factors. Firstly, as the number of spheres increases, the effective size of the metal (Au) area in the Janus particles also increases. This results in a shift in the plasmonic resonance towards longer wavelengths, away from the excitation wavelength used in Raman spectroscopy. Consequently, the efficiency of plasmonic enhancement decreases, leading to a decrease in Raman signal intensity. Secondly, when the particles come into proximity to each other, either through aggregation or touching, the plasmonic coupling between them becomes significant. This proximity results in a reduction of the gap between the particles, which is critical for enhancing the local electric field and Raman scattering. However, as the particles touch each other, the gap between them diminishes, leading to a decrease in the plasmonic enhancement effect and consequently a decrease in the Raman signal intensity.

 The decrease in Raman intensity as the number of spheres increases has been confirmed by utilizing a finite-difference-time-domain (FDTD) approach to validate the experimental results. To prove this, we modeled three cases of 1, 2, and 3 Janus particles separately, and after the simulations, we calculated the electromagnetic field enhancement distributions at a certain wavelength of light source. From the electromagnetic fields for all the cases, we derived the maximum values for each case and plotted them to compare.

**Fig. 6:**
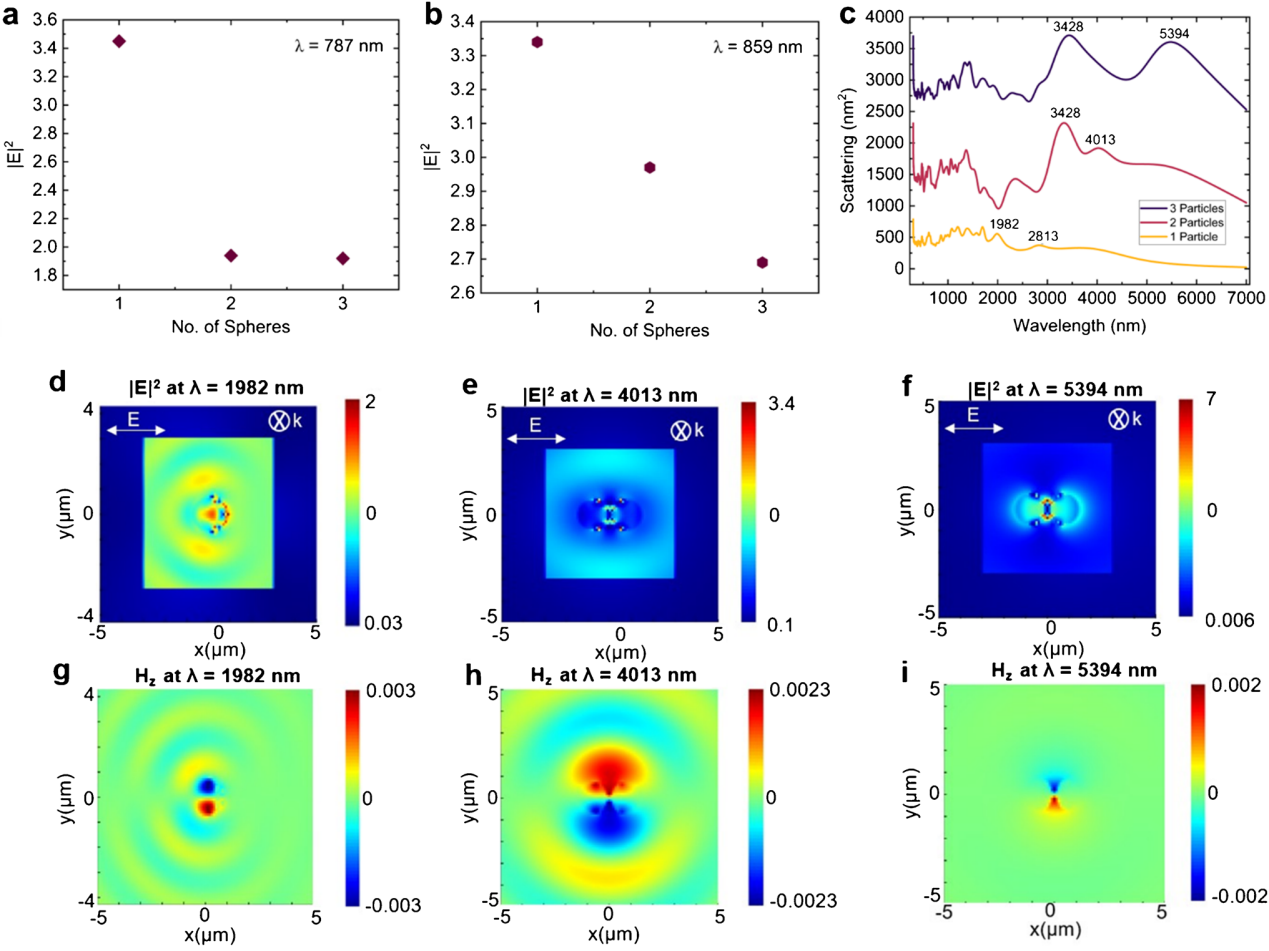
Variation of the maximum electric field with the number of Janus particles (JPs) at **a** λ = 787 nm and **b** λ = 859 nm. **c** Scattering cross-section with characteristic peaks for assemblies of one, two, and three JPs. Electric field distribution for **d** one, **e** two, and **f** three JPs at their respective characteristic scattering peaks: λ = 1982 nm for one JP, λ = 4013 nm for two JPs, and λ = 5394 nm for three JPs. The corresponding magnetic field distributions at these wavelengths are shown for **g** one, **h** two, and **i** three JPs

**Fig. 7:**
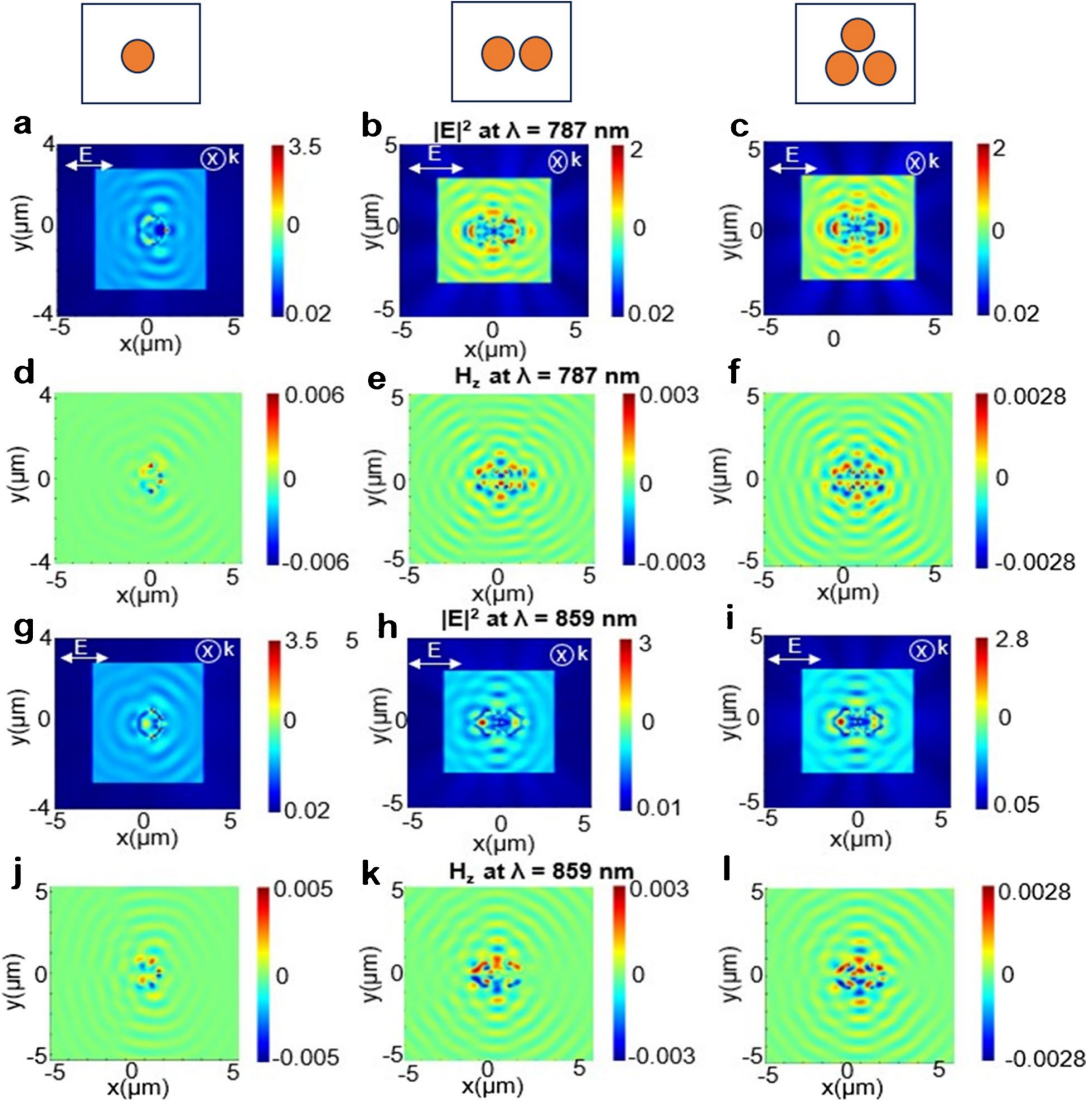
Electric and magnetic field distributions for Janus particles (JPs) at different wavelengths. **a**–**c** Electric field distribution for one, two, and three JPs at λ = 787 nm. **d**–**f** Corresponding magnetic field distribution at λ = 787 nm. **g**–**i** Electric field distribution for one, two, and three JPs at λ = 859 nm. **j**–**l** Corresponding magnetic field distribution at λ = 859 nm. Field intensity is represented by color, with blue indicating low magnitude and red indicating high magnitude. The incident electric field is polarized along the X-direction, while the propagation vector (k) is oriented along the Z-direction

To validate our experimental results shown in Fig. 5b, we performed an electromagnetic simulation using FDTD. Figure 6a and b shows the maximum electric field intensity variation with the number of JPs at λ = 787 nm and 859 nm. In our Raman experiments, we employed the laser with an excitation wavelength of 785 nm, and the characteristic Raman peak was plotted at a wavenumber of 1002 cm^−1^ (Fig. 5d, e). That is why we choose to plot the electric field intensities at these two wavelengths (Raman shift of 1002 cm^−1^ for an excitation wavelength of 785 nm corresponds to ~ 852 nm). The color shows the strength of the electric field with blue signifies low and red indicated high electric field. The incident electric field was polarized in the X-direction, and the propagation vector (k) was in the Z-direction. The FDTD simulations showed a similar variation like the experimental results—with the increase of JPs the electric field decreased. Since, Raman intensity, ${I}_{\text{Raman}}={I}_{\text{Excitation}}\times {I}_{\text{Emission}}$, we observed a decrease in the Raman intensity with increase in the number of JPs as both excitation electric field (at λ = 787 nm) and Raman emission electric field (at λ = 859 nm) decreased with the increase of number of JPs (Fig. 6a, b). Figure 6c shows the scattering spectra of the JPs (one, two, and three) plotted over wavelengths ranging from 300 to 7000 nm. The characteristic peaks appeared mostly in IR-range. That could be the reason we did not observe any Raman enhancement with laser excitation of λ = 785 nm. The electric field for the JPs at their characteristic scattering peaks in the IR-range is shown in Fig. 7d–f. The corresponding magnetic field variations are shown in Fig. 7g–i. The electric field was the strongest for the three JPs case at λ = 5394 nm (Fig. 6f). The plasmonic fields for single and three JPs are confined strongly within the particles (Fig. 6g, i), whereas for two JPs, the plasmonic field extends beyond the particle dimensions (Fig. 6h), indicating better coupling with surrounding molecules at λ = 4013 nm.

Figure 7 presents the electric and magnetic field distributions for Janus particles (JPs) at the Raman excitation wavelength (λ = 787 nm) and near the Raman emission wavelength (λ = 859 nm), corresponding to the Raman peak at 1002 cm⁻^1^. The highest electric field intensity was observed for the single JP case at both excitation and emission wavelengths. The electric field was primarily localized around the JP surfaces (Fig. 7a–c, g–i). The plasmonic coupling strength, represented by the Hz magnitude, was also highest for the single JP (Fig. 7d, j), decreasing for two JPs (Fig. 7e, k) and three JPs (Fig. 7f, l).

The Derjaguin-Landau-Verwey-Overbeek (DLVO) theory provides a fundamental framework for understanding the aggregation behavior of colloidal particles [57], including Janus particles, by considering the balance between attractive van der Waals forces and repulsive electrostatic interactions. For Janus particles, the inherent asymmetry in their surface chemistry and charge distribution introduces unique variations in these forces, influencing their aggregation dynamics. In particular, the localized differences in surface charge or functional groups on Janus particles can lead to anisotropic interaction [44], where one hemisphere may promote attraction while the other resists it, resulting in directional aggregation or specific assembly patterns. The pH of the surrounding medium plays a critical role in modulating the electrostatic potential of the Janus particle surfaces, altering the Debye length and the strength of repulsive forces [9, 11]. This sensitivity to environmental conditions allows for tunable aggregation behavior that deviates from traditional colloidal systems described by symmetric DLVO theory. Understanding these nuanced interactions is crucial for leveraging Janus particle aggregation in targeted applications, such as stimuli-responsive materials and engineered self-assembly.

## Conclusions

We successfully synthesized micrometer-sized JPs by depositing gold onto polystyrene spheres and evaluated two distinct polyethylene glycol (PEG) functionalization strategies to mitigate non-specific aggregation while facilitating interactions with lysozyme. Raman spectroscopy confirmed successful protein conjugation and demonstrated the pH-responsive behavior of the JPs, exhibiting reversible aggregation below pH 6 and disaggregation above pH 7. To further characterize their plasmonic properties, we performed surface-enhanced Raman spectroscopy (SERS) experiments in the visible wavelength range, demonstrating that JP aggregation significantly modulates Raman signal intensity. Raman mapping revealed the spatial distribution of the electromagnetic field for both individual and aggregated JPs, highlighting enhanced field localization in the aggregated state. Additionally, finite-difference time-domain (FDTD) simulations provided mechanistic insights into the observed Raman modulation by quantifying the localized electric field enhancement. Looking ahead, advanced techniques such as high-resolution TEM [58] can reveal atomic-level details of the assembled structures, while 4D-STEM has been shown to map dynamic 3D crystallographic changes during aggregation [59, 60]. In addition, scattering spectroscopy methods—for example, dynamic light scattering and related real-time techniques [61]—offer the capability to monitor aggregation kinetics as particle sizes and optical properties evolve, and high-speed imaging with fast detectors [62] can capture rapid self-assembly events. Together, these methods promise to provide deeper insights into aggregation kinetics and enable more precise control over the self-assembly process. The demonstrated pH-responsive behavior suggests that these JPs have potential applications in controlled drug delivery, diagnostics, and biosensing. Future work could determine precise aggregation kinetics under different pH conditions and explore tunability in other physiological environments to expand their applicability in biomedical and sensing technologies.

## Supplementary Information

Below is the link to the electronic supplementary material.

ESM 1 (DOCX 476 KB)

ESM 2 (PPTX 3.81 MB)

## Data Availability

The datasets will be available from the corresponding author on reasonable request.
