# Supplementary material for: pH-tuned reversible self-assembly of Janus particles for enhanced Raman imaging and sensing
Source: Anal Bioanal Chem. 2025 May 3;417(30):6755–67. doi: 10.1007/s00216-025-05887-z (PMC12680853; doi:10.1007/s00216-025-05887-z)
Supplement: Supplementary file 1 — (DOCX 476 KB) [file 216_2025_5887_MOESM1_ESM.docx]

**Supporting Information**

**pH-Tuned Reversible Self-Assembly of Janus Particles for Enhanced Raman Imaging and Sensing**

**Maria Iftesum^1, ‖^, Mohan Kumar Dey^1,†^, Alisha Prasad^2,†^, Jin Gyun Lee^3,‖^, Ram Devireddy^1^, Bhuvnesh Bharti^3^, Manas Ranjan Gartia^1,^***

^1^Department of Mechanical and Industrial Engineering, Louisiana State University, Baton Rouge, LA 70803 USA

^2^Catalent Pharma, St. Petersburg, FL 33716 USA

^3^Cain Department of Chemical Engineering, Louisiana State University, Baton Rouge, LA 70803 USA

^†, ‖^Equal contribution authors

*Corresponding author: [mgartia@lsu.edu](mailto:mgartia@lsu.edu)

**Table S1.** Raman peak positions for the different components of the Janus Particles

|  | **PS** | **11-MUA** | **EDC** | **Lysozyme** | **Au + PS** | **Au + PS + MUA** | **Au + PS + MUA + EDC** | **Au + PS + MUA + EDC + Lys** |
| --- | --- | --- | --- | --- | --- | --- | --- | --- |
| 450 - 550 |  |  |  | 471, 527 |  |  |  |  |
| 551 - 650 | 622 |  |  | 622, 648 | 624 |  | 624 | 624 |
| 651 - 750 |  | 738 |  |  |  |  |  |  |
| 751 - 850 | 798 |  | 827 | 755, 798 | 793 |  |  |  |
| 851 - 950 |  | 909 |  |  |  |  |  |  |
| 951 - 1050 | 1005, 1029 |  |  |  | 1004, 1033 | 1004, 1033 | 1004, 1033 | 1004, 1033 |
| 1051 - 1150 |  | 1060, 1107 | 1149 | 1122 |  | 1197 |  | 1191 |
| 1151 - 1250 | 1157, 1205 |  |  |  | 1201 |  | 1203 |  |
| 1251 - 1350 |  | 1290 |  | 1255 |  |  |  |  |
| 1351 - 1450 |  | 1436 |  |  |  |  |  |  |
| 1451 - 1550 | 1452 | 1457 | 1454 |  | 1451 |  |  |  |
| 1551 - 1650 | 1603 | 1631 | 1642 | 1551 | 1603 | 1605 | 1602 | 1604 |
| 1651 - 1750 |  |  |  | 1715 |  |  |  |  |
| 1751 - 1850 |  |  |  |  |  |  |  |  |
| 1851 - 1950 |  |  |  | 1883 |  |  |  |  |
| 1951 - 2050 |  |  |  |  |  |  |  |  |
| 2051 - 2150 |  |  |  |  |  |  |  |  |
| 2151 - 2250 |  |  |  |  |  |  |  |  |
| 2251 - 2350 |  |  |  |  |  |  |  |  |
| 2351 - 2450 |  |  |  |  |  |  |  |  |
| 2451 - 2550 |  | 2549 |  |  |  |  |  |  |
| 2551 - 2650 |  | 2575 |  |  |  |  |  |  |
| 2651 - 2750 |  | 2722 |  |  |  |  |  |  |


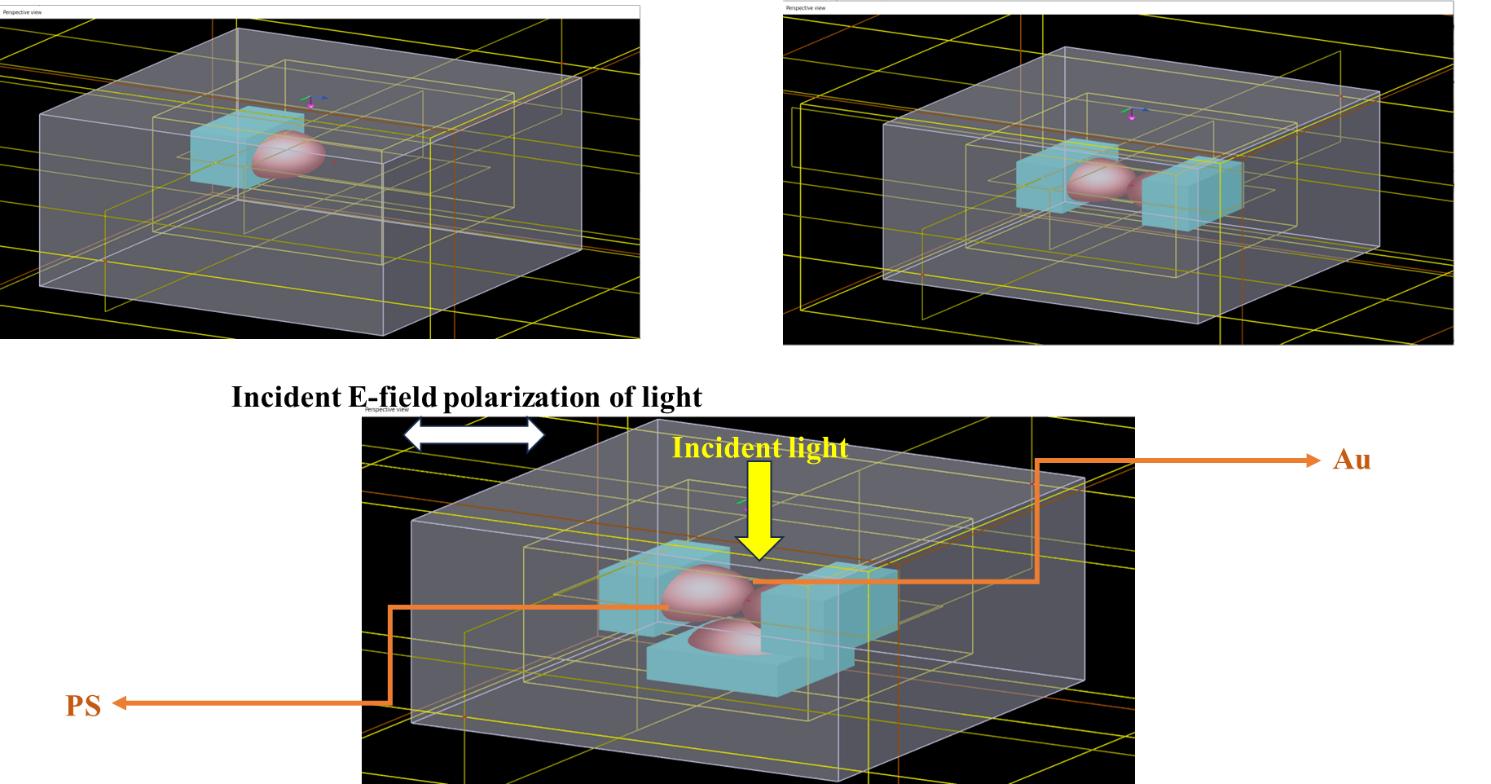


**Figure S1.** Schematic of FDTD Simulation for one, two, and three Janus particles using Ansys Lumerical software.
