# Supplementary material for: pH-tuned reversible self-assembly of Janus particles for enhanced Raman imaging and sensing
Source: Anal Bioanal Chem. 2025 May 3;417(30):6755–67. doi: 10.1007/s00216-025-05887-z (PMC12680853; doi:10.1007/s00216-025-05887-z)
Supplement: Supplementary file 2 — (PPTX 3.81 MB) [file 216_2025_5887_MOESM2_ESM.pptx]

## Slide 1
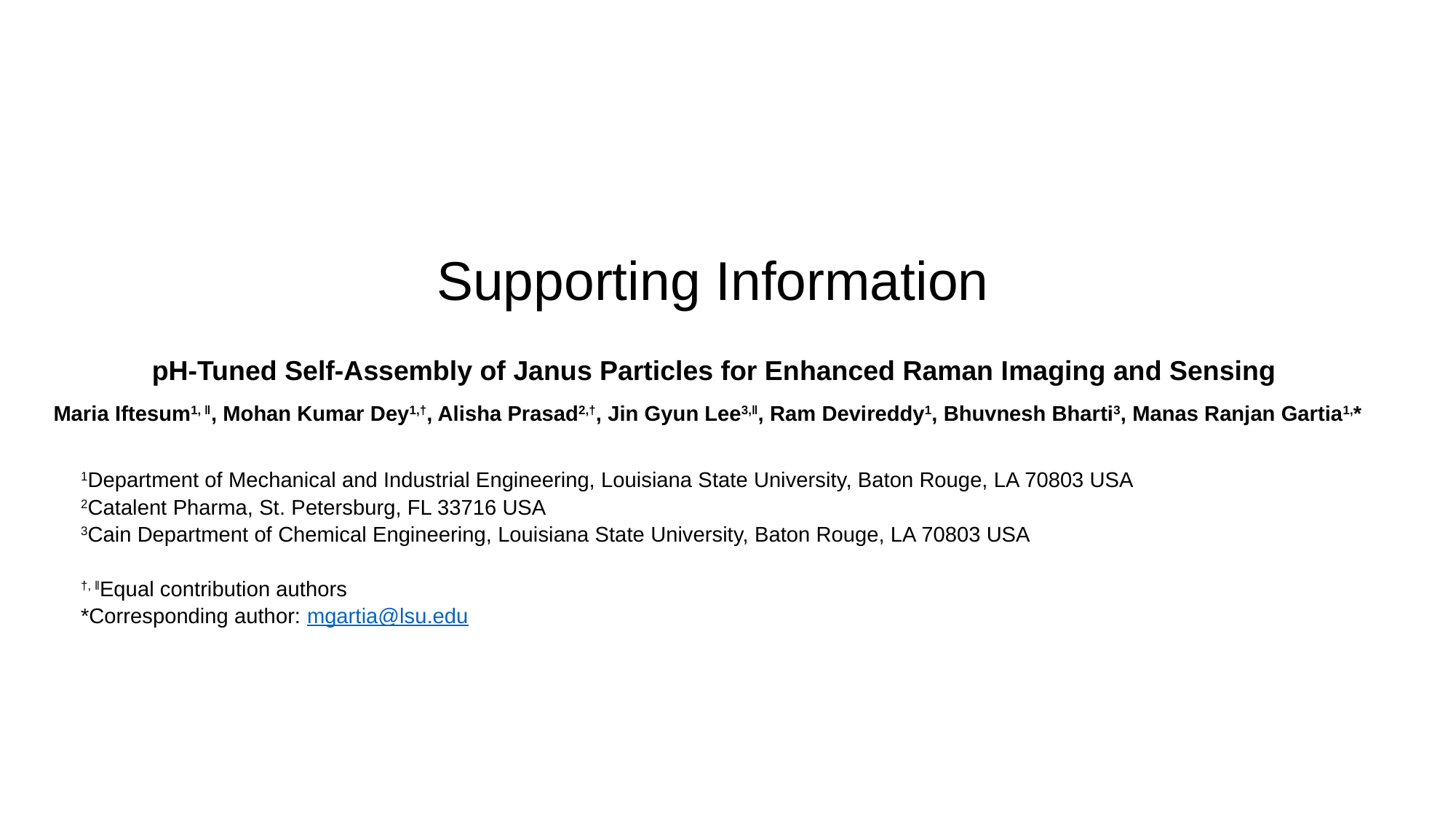

Supporting Information
pH-Tuned Self-Assembly of Janus Particles for Enhanced Raman Imaging and Sensing
Maria Iftesum1, ‖, Mohan Kumar Dey1,†, Alisha Prasad2,†, Jin Gyun Lee3,‖, Ram Devireddy1, Bhuvnesh Bharti3, Manas Ranjan Gartia1,*
1Department of Mechanical and Industrial Engineering, Louisiana State University, Baton Rouge, LA 70803 USA
2Catalent Pharma, St. Petersburg, FL 33716 USA
3Cain Department of Chemical Engineering, Louisiana State University, Baton Rouge, LA 70803 USA
†, ‖Equal contribution authors
*Corresponding author: mgartia@lsu.edu

## Slide 2
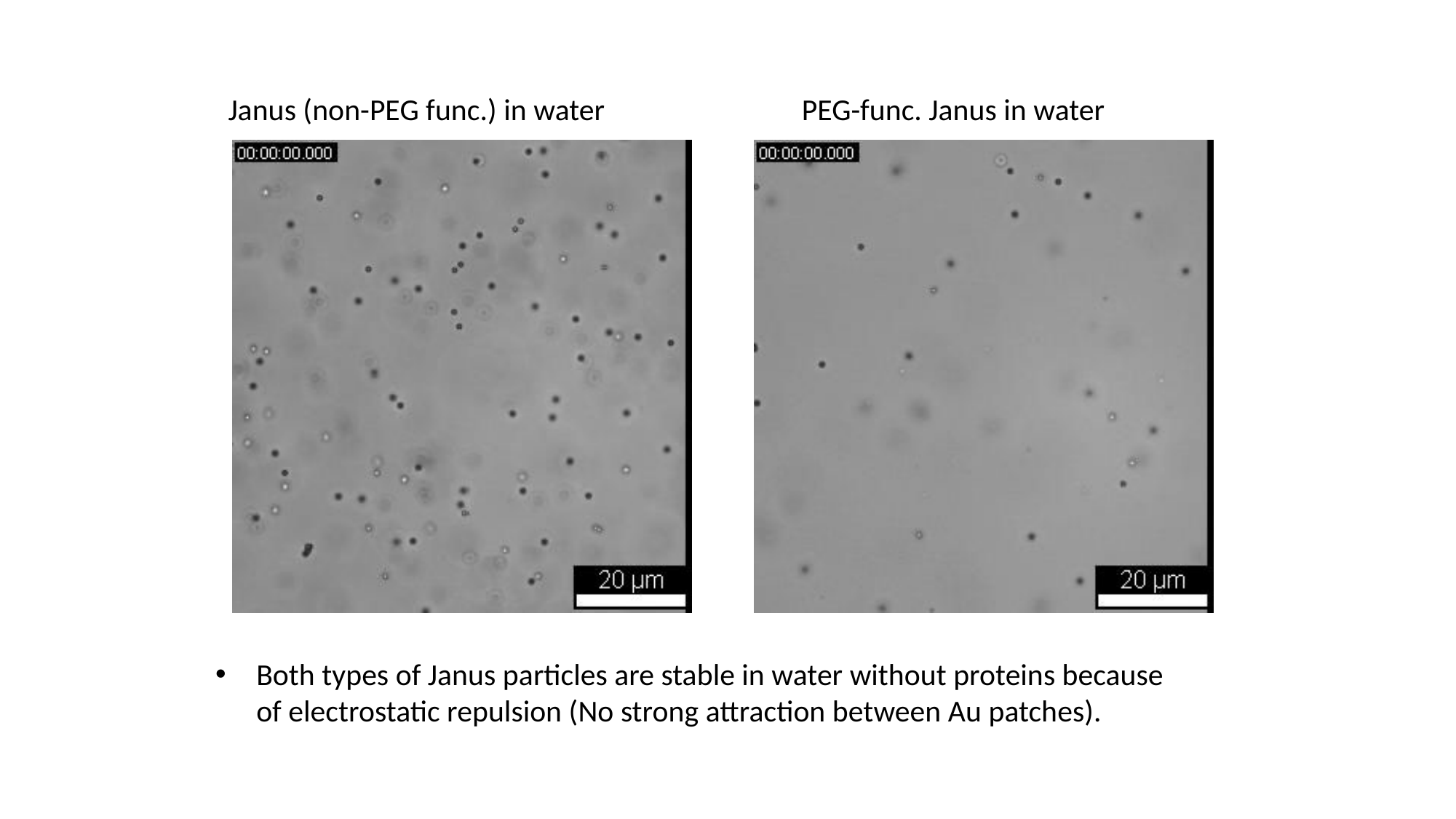

Janus (non-PEG func.) in water
PEG-func. Janus in water
Both types of Janus particles are stable in water without proteins because of electrostatic repulsion (No strong attraction between Au patches).

## Slide 3
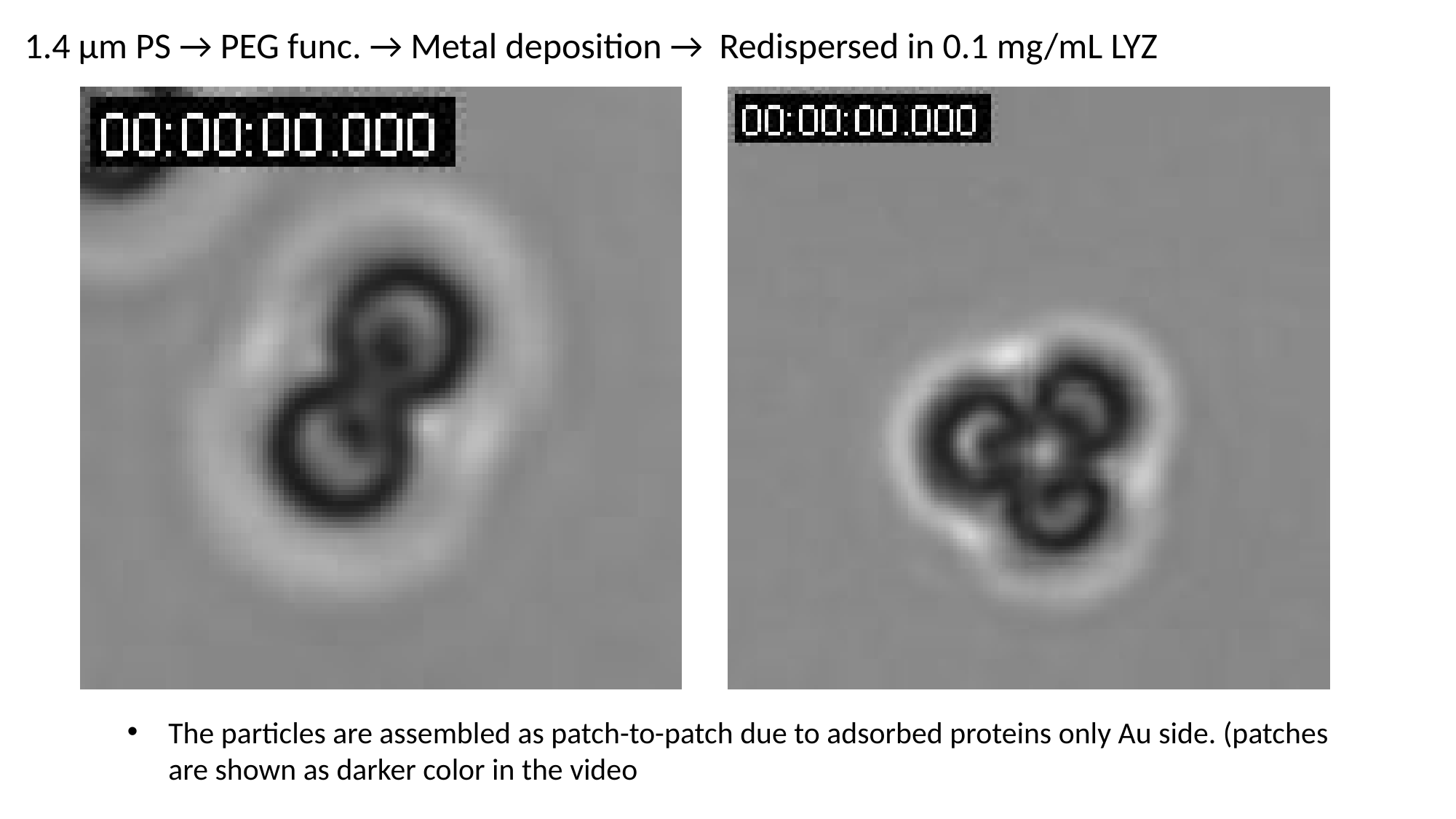

1.4 µm PS → PEG func. → Metal deposition → Redispersed in 0.1 mg/mL LYZ
The particles are assembled as patch-to-patch due to adsorbed proteins only Au side. (patches are shown as darker color in the video

## Slide 4
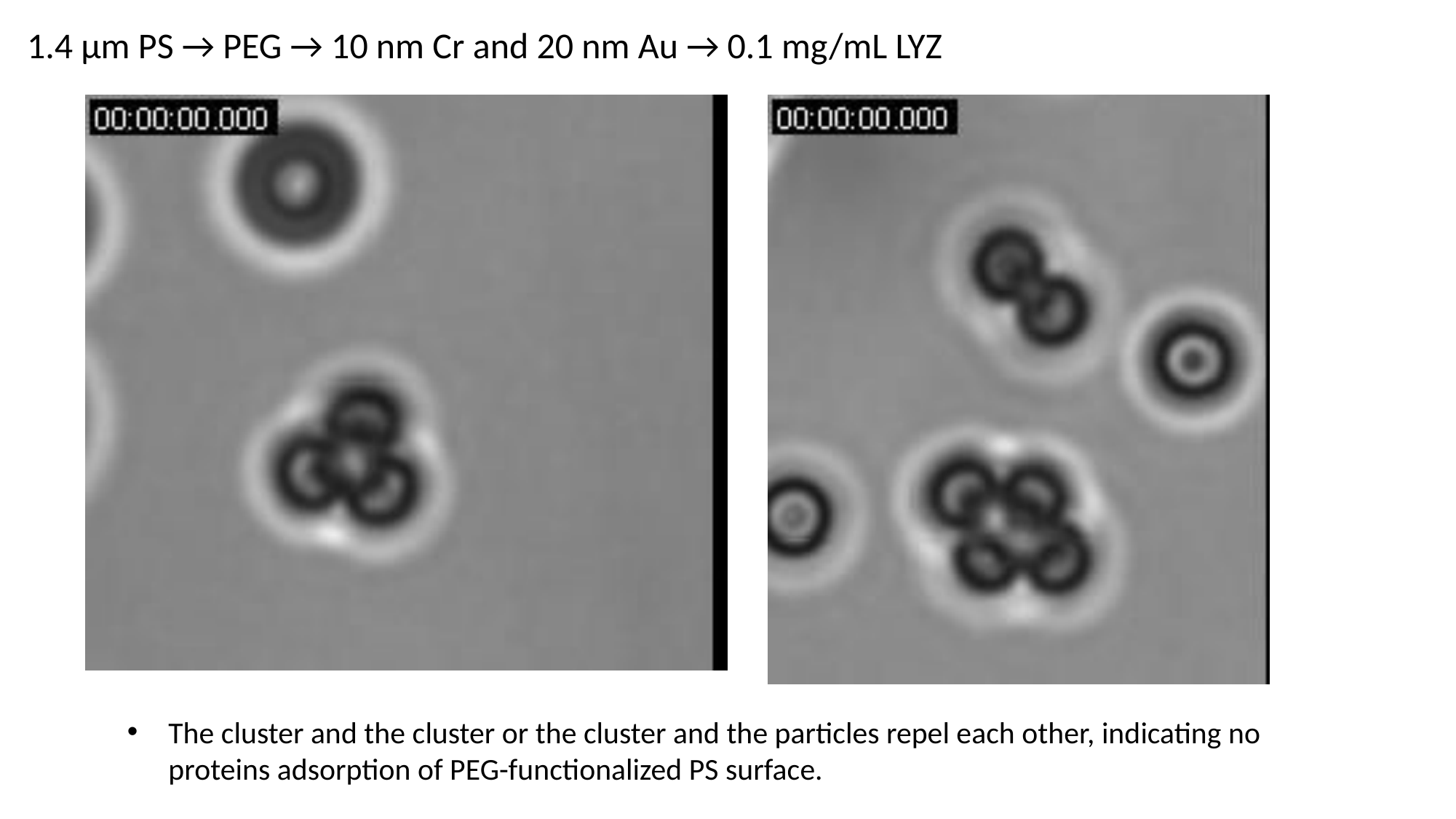

1.4 µm PS → PEG → 10 nm Cr and 20 nm Au → 0.1 mg/mL LYZ
The cluster and the cluster or the cluster and the particles repel each other, indicating no proteins adsorption of PEG-functionalized PS surface.

## Slide 5
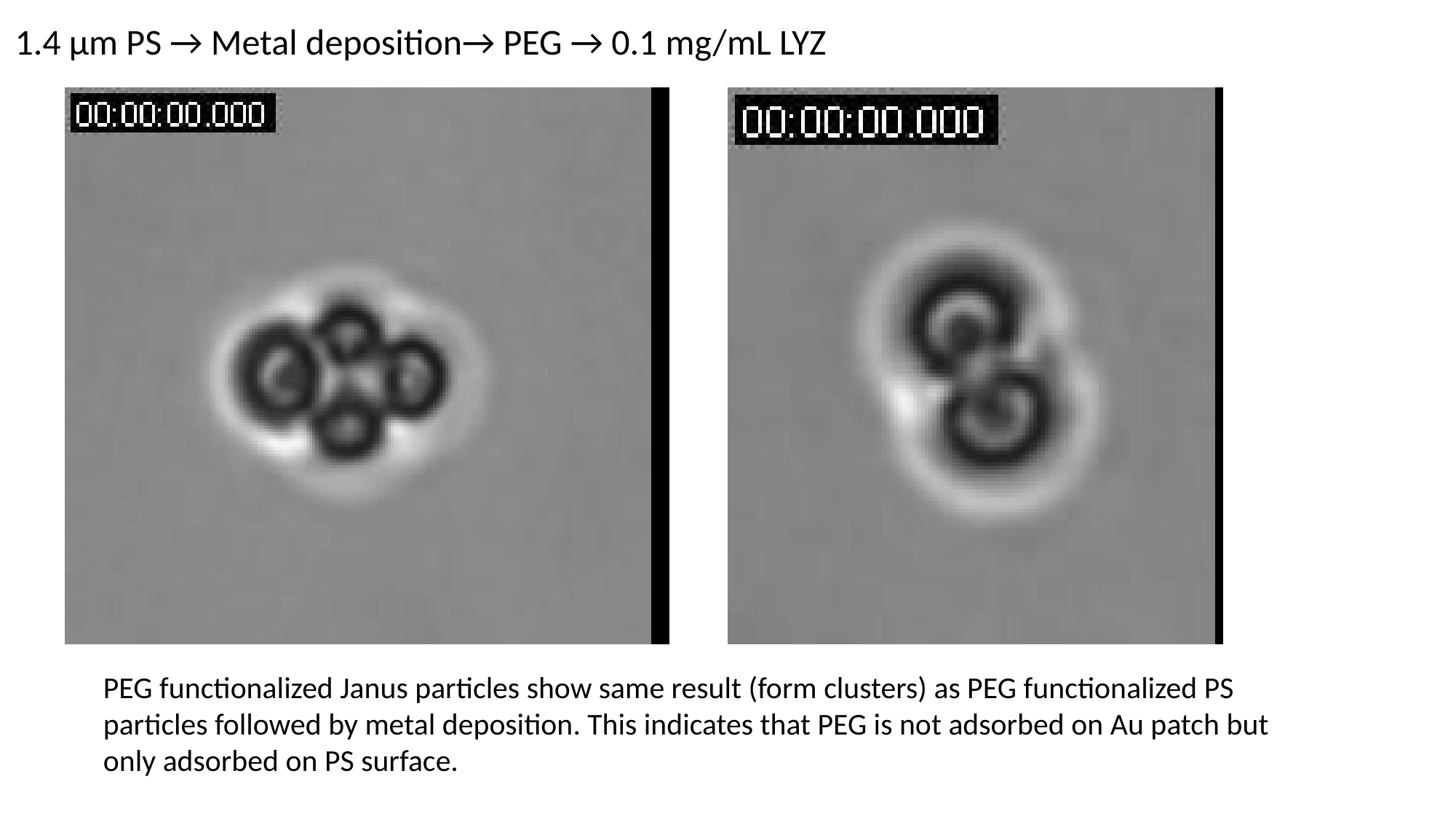

1.4 µm PS → Metal deposition→ PEG → 0.1 mg/mL LYZ
PEG functionalized Janus particles show same result (form clusters) as PEG functionalized PS particles followed by metal deposition. This indicates that PEG is not adsorbed on Au patch but only adsorbed on PS surface.

## Slide 6
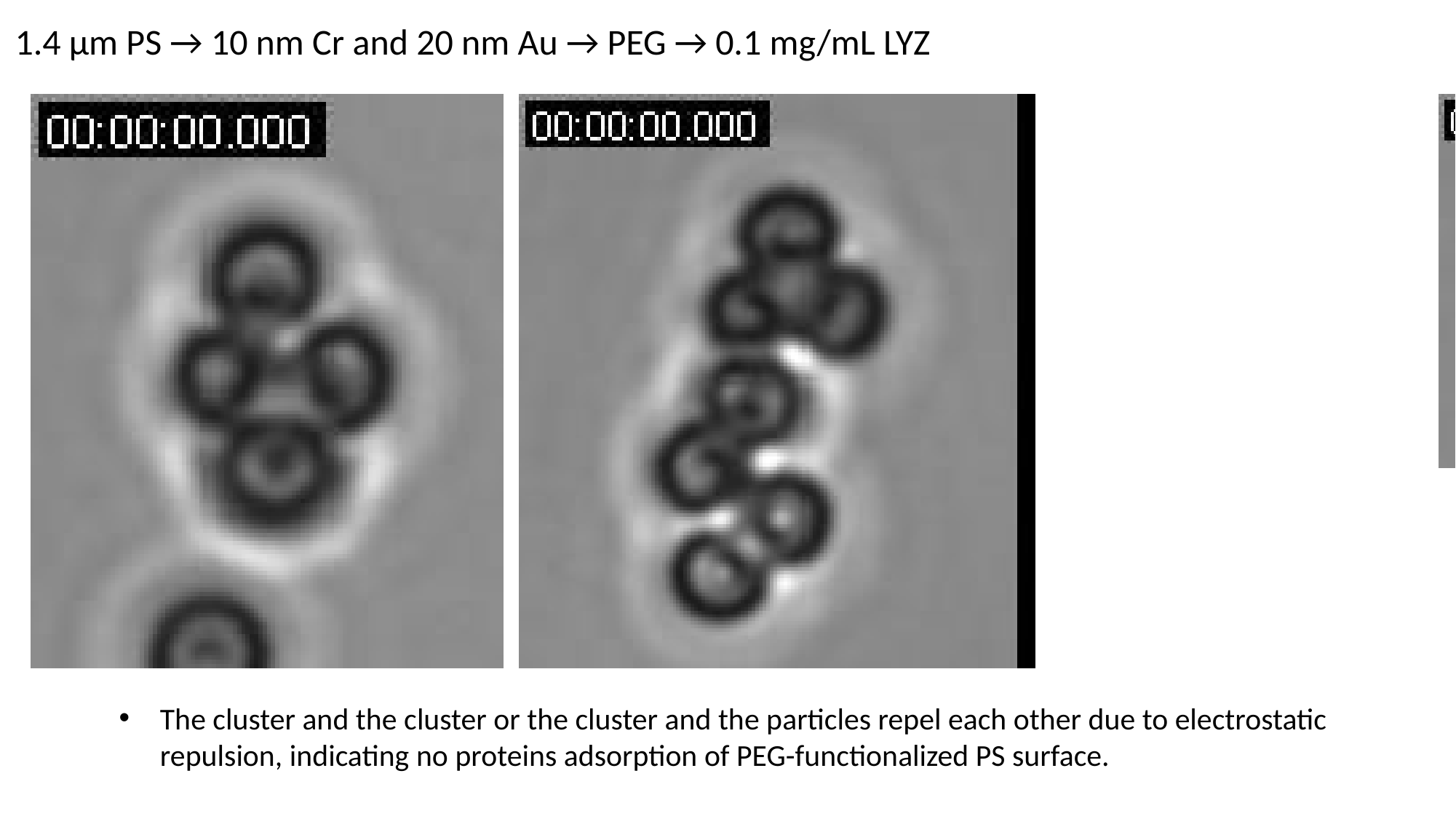

1.4 µm PS → 10 nm Cr and 20 nm Au → PEG → 0.1 mg/mL LYZ
The cluster and the cluster or the cluster and the particles repel each other due to electrostatic repulsion, indicating no proteins adsorption of PEG-functionalized PS surface.
